# Utilizing liposomal encapsulation approach to address nephrotoxic challenges of colistimethate sodium through a preclinical study

**DOI:** 10.3389/fphar.2023.1282464

**Published:** 2023-11-22

**Authors:** Raktham Mektrirat, Noppanut Paengjun, Peerawit Chongrattanameteekul, Sonthaya Umsumarng, Suppara Cheunsri, Kornravee Photichai, Kittima Lewchalermvong, Chalutwan Sansamur, Siriporn Okonogi, Wasan Katip

**Affiliations:** ^1^ Department of Veterinary Bioscience and Public Health, Faculty of Veterinary Medicine, Chiang Mai University, Chiang Mai, Thailand; ^2^ Research Center for Veterinary Biosciences and Veterinary Public Health, Faculty of Veterinary Medicine, Chiang Mai University, Chiang Mai, Thailand; ^3^ Center of Excellence in Pharmaceutical Nanotechnology, Faculty of Pharmacy, Chiang Mai University, Chiang Mai, Thailand; ^4^ Center of Veterinary Diagnosis and Technology Transfer, Faculty of Veterinary Medicine, Chiang Mai University, Chiang Mai, Thailand; ^5^ School of Agricultural Technology, King Mongkut’s Institute of Technology Ladkrabang, Bangkok, Thailand; ^6^ Akkhraratchakumari Veterinary College, Walailak University, Nakhon Si Thammarat, Thailand; ^7^ Centre for One Health, Walailak University, Nakhon Si Thammarat, Thailand; ^8^ Department of Pharmaceutical Sciences, Faculty of Pharmacy, Chiang Mai University, Chiang Mai, Thailand; ^9^ Department of Pharmaceutical Care, Faculty of Pharmacy, Chiang Mai University, Chiang Mai, Thailand

**Keywords:** acute kidney injury, antimicrobial, cytotoxicity, liposome, rat, symmetric dimethylarginine

## Abstract

The use of Colistin, a last-resort antimicrobial drug, carries the risk of acute kidney injury. The objective of the study was to assess the effectiveness of colistin-encapsulated liposomes (CL) in reducing nephrotoxicity. Additionally, a liposomal preparation of colistimethate sodium was formulated using the reverse phase evaporation method with a 3:1 ratio of phospholipids to cholesterol. The liposomal properties were evaluated using scanning electron microscopy, photon correlation spectroscopy, and release kinetic assay. The killing kinetics of the formulations on embryonic kidney cells were assessed using *in vitro* MTT reduction assay. The nephrotoxicity of CL and colistimethate sodium solution (CS) was evaluated *in vivo* by administering a dose of 20 mg/kg to rats every 12 h for 3 days, with a negative control group receiving a 0.9% saline solution (NSS). The study results revealed that monodisperses of CL showed a smooth surface and distinct boundaries, with an average size of 151.50 ± 0.46 nm and a narrow size distribution of 0.25 ± 0.01. The liposomal particles showed high entrapment efficiency of 96.45% ± 0.41%, with a ζ-potential of −60.80 ± 1.01 mV and a release rate of 50% of colistimethate sodium within the first 480 min. The CL induced nephrocytotoxicity in a concentration- and time-dependent manner. However, CS had notably lower IC_50_ values compared to its liposome preparations at 48 and 72 h (*p <* 0.05). *In vivo* study results show that serum levels of symmetric dimethylarginine (SDMA) and total white blood cell count (WBC) were significantly lower in the CL group (SDMA = 8.33 ± 1.70 μg/dL; WBC = 7.29 ± 0.99 log_10_ cells/mL) compared to the CS group (SDMA = 15.00 ± 1.63 μg/dL; WBC = 9.73 ± 0.51 log_10_ cells/mL). Our study findings enhance the understanding of the safety profile of CL and its potential to improve patient outcomes through the use of liposomal colistin medication. Additional clinical studies are necessary to establish the optimal safety regiment in humans.

## Introduction

Following the widespread usage of broad-spectrum antimicrobial drugs, the prevalence of multidrug resistance (MDR) in bacterial infections is currently a leading cause of morbidity and mortality on a global scale. Moreover, the bacterial production of plasmid-mediated ESBLs limits the number of antimicrobials that can be used to treat infections successfully. Whereas the resistance to last-resort antibiotics is particularly concerning because it might soon restrict the use of antimicrobial therapy for bacterial serious diseases (Fair and Tor, 2014). Due to a lack of other choices, colistin (also known as polymyxin E) has resurfaced as a last-line of defense against multidrug-resistant Gram-negative bacteria (Li et al., 2006). Guidelines for the most effective clinical use of colistin treatment are provided by international consensus recommendations (Tsuji et al., 2019). For the treatment of serious multidrug-resistant Enterobacterales, *Pseudomonas aeruginosa*, *Acinetobacter baumannii* infections, intravenous colistin has been performed. However, it is most commonly utilized in serious clinical conditions including sepsis, and also pneumonia related with mechanical ventilation (VAP) in the intensive care unit (ICU).

Unfortunately, clinical use of colistin has been limited due to its potential to cause acute kidney injury (Falagas and Kasiakou, 2006; Hartzell et al., 2009; Arrayasillapatorn et al., 2021; Ustundag et al., 2022). Colistin can induce acute kidney injury via several potential mechanisms, including direct tubular toxicity, oxidative stress, and disruption of cell membrane integrity. Factors such as pre-existing renal disease and the concomitant use of other nephrotoxic medications can further increase the risk of acute kidney injury (AKI) (Jafari and Elyasi, 2021). Recently developed clinical practice guidelines from the kidney disease community have highlighted the importance of monitoring kidney function in patients treated with colistin to prevent and manage its nephrotoxicity (Pogue et al., 2017; Shields et al., 2017; Eljaaly et al., 2021). Laboratory studies using cell-based assay and animal models have extensively reported colistin-induced nephrotoxicity, providing insights into the mechanisms underlying its toxicity (Keirstead et al., 2014; Heybeli et al., 2019). These laboratory findings align with clinical concerns, suggesting a need for greater awareness of the risks associated with colistin treatment and a greater understanding of the mechanisms of AKI to improve patient outcomes. Ultimately, this highlights the importance of a collaborative effort between clinical and laboratory approaches to better understand and manage the risks of colistin-induced nephrotoxicity.

Liposomes are a promising drug delivery system that could mitigate the toxicity associated with colistin use (Ferreira et al., 2021; Zong et al., 2022). Since, the liposomes are small spherical vesicles consisting of a phospholipid bilayer that can encapsulate and deliver drugs to target cells. They have been shown to improve drug efficacy, reduce toxicity, and enhance drug stability (Bozzuto and Molinari, 2015; Faustino and Pinheiro, 2020). Recent developments in liposomal colistin have shown promising results in animal models and clinical trials, animal models have been extensively used to evaluate the efficacy of liposome-based drug delivery systems in enhancing the therapeutic efficacy of antimicrobials while reducing their toxic side effects (Alarfaj et al., 2022; Joshi et al., 2023). Several clinical trials have also been conducted to evaluate the efficacy of liposome-based drug delivery systems in reducing the occurrence of antimicrobial-induced nephrotoxicity (Bulbake et al., 2017; Zong et al., 2022). The aim of this study was to evaluate the renal cytotoxicity of colistimethate sodium, as well as to assess the safety of colistin liposome formulations (CL) in reducing its toxic side effects, particularly in relation to nephrotoxicity in a rat model.

## Materials and methods

### Cell lines and culture condition

A human embryonic kidney cell line (Name, 2A; Number, CRL-12013) was purchased from American Type Culture Collection (ATCC, Manassas, VA, United States). The culture plates of human embryonic kidney cells were adjusted to 4 × 10^4^ cells/well in RPMI 1640 supplemented with 1% heat inactivated fetal bovine serum (FBS) (Gibco, Waltham, MA, United States) and 1% penicillin/streptomycin (Sigma-Aldrich, St. Louis, MO, United States) with a final concentration of 100 units per ml. The cells were maintained at 37°C and 5% CO_2_ in a humidified incubator (Thermo Fischer Scientific, San Jose, CA, United States).

### Dose-response nephrocytoxicity test of colistimethate sodium

The culture plates of human embryonic kidney cells were treated with 0–200 μg/mL colistimethate sodium (Able Medical Co.,Ltd., Mahasarakham, Thailand) and incubated in 5% CO_2_ incubator under humidified conditions at 37°C. The cell viability was measured by MTT [3-(4,5-dimethylthiazol-2-yl)-2,5-diphenyl tetrazolium bromide] reduction assay at 24 h after exposure of the tested solution. The volume of 10 µL of 5 mg/mL MTT was added into 200 µL of cell suspension and incubated for 4 h The volume of 100 µL 0.1 M HCl in absolute isopropanol was added after incubation. The colorimetric determination of formazan product was spectrophotometrically measured at 570 nm. Cell viability was expressed as a percentage of the control culture with normal saline solution. This experiment was done with four independent replications.

### Preparation of colistin-encapsulated liposome

The CL was prepared using the reverse phase evaporation method with modifications as described previously (Shi and Qi, 2018). First, a mixture of exact phospholipid and cholesterol at various molar ratios in methanol was vortex-mixed for 30 s. The lipid solution was then evaporated under vacuum at 40°C for 20 min using a rotary evaporator. Next, a solution of colistimethate sodium in normal saline was mixed with the exact amount of diethyl ether before being added to the lipid film. The final mixture was then sonicated until a stable emulsion formed. The organic solvent was removed by using a rotary evaporator until a film was formed. The film was sonicated with 10 mL of normal saline at 40°C for 10 min to create the CL suspension. Finally, unentrapped colistimethate sodium was removed using a mini-column centrifugation method employing a Sephadex G-50 column (Sigma-Aldrich, St. Louis, MO, United States). The experiment was conducted thrice, with a fresh syringe packed with gel for each repetition.

### Visualization of surface liposomal morphology

The specimen was freeze-dried, sputter-coated with gold particles, and then examined for its cell morphological appearance under a field-emission scanning electron microscope (FE-SEM) (JSM-6335F, JEOL Ltd., Tokyo, Japan).

### Physicochemical characterization of the formulation

The present investigation concerns the comprehensive examination of the internal phase droplets diluted in Millipore water at a ratio of 1:100 (v/v), focusing on the measurement of particle size, size distribution, and ζ-potential. Photon correlation spectroscopy (PCS) was employed as the primary technique for this purpose, utilizing a Zetasizer Nano ZS instrument (Malvern Instruments Ltd., Malvern, Worcestershire, United Kingdom) at a temperature of 25°C. To determine the particle size and size distribution of each mixture, the sample was transferred into a cuvette and measured on a fixed angle of 173°. The particle size was expressed as an average diameter in nm, while the particle size distribution was expressed as polydispersity index (PdI). For the determination of ζ-potential, each mixture was transferred into DT51070 folded capillary cells (Malvern, Worcestershire, United Kingdom) prior to being subjected to PCS. The ζ-potential of the samples was automatically calculated based on the Smoluchowski equation (Rizvi and Saleh, 2018) using the Zetasizer software version 7.1 (Malvern, Worcestershire, United Kingdom). All experiments were conducted in triplicate in order to ensure the reliability of the obtained results.

### Determination of liposomal entrapment efficiency

The measurement of entrapment efficiency for CL was conducted using the mini-column centrifugation technique, following the procedures outlined in previous reports (Torchilin and Weissig, 2003). Briefly, the mini-columns were created by utilizing 15 mL plastic syringe barrels filled with Sephadex G-50 (Sigma-Aldrich, St. Louis, MO, United States). To remove excess fluid from the Sephadex beads, centrifugation was performed at 3,000 rpm at 25°C for 3 min. Next, 0.4 mL of CL suspension was added to the column beds and centrifuged at 1,500 rpm at 25°C for 3 min. Following this step, the columns were washed twice with 0.2 mL of distilled water. The elutes containing CL were collected and digested, and the resulting clear solution was then analyzed using high-performance liquid chromatography (HPLC).

### Colistin-encapsulated liposome release kinetic assay

The mixed solutions of phosphate-buffered saline (PBS, pH 7.4) were used as the *in vitro* release media. An aliquot of 4 mL of liposomal preparation was mixed with 16 mL of PBS and gently stirred (200 rpm) at 37°C. A 1 mL sample was withdrawn to determine the total drug content, while an additional 0.5 mL sample was mixed with Triton X-100 (LOBA Chemie Pvt. Ltd., MB, India) and centrifuged at 12,000 rpm for 10 min. After centrifugation, 0.1 mL of the supernatant was collected and diluted with 0.9 mL. The diluted sample was then transferred to nylon membrane (0.22 µm) ultra-centrifugation filters for the determination of free drug content using HPLC with Prominence-i (LC-2030) (Shimadzu, Kyoto, Japan). Three replicates of each sample were evaluated at each time point for 0, 5, 120, 240, 480, 720, and 1,440 min.

### Time-dependent nephrocytoxicity test of the formulation

The killing kinetic of the formulations was performed to determine the time killing rates on human embryonic kidney cells. The cells were inoculated into wells of a 96-well plate at a density of 4 × 10^4^ cells/well. The experiment was performed in four independent replicates in the culture plates administered the formulations of CS and CL. The culture plates were incubated at 37°C in a 5% CO_2_ incubator for 48 and 72 h. The cell viability was measured by MTT reduction assay as described above.

### Animal preparation and ethical approval

Twelve male Sprague Dawley rats (*Rattus norvegicus*) were procured from Nomura Siam International Co., Ltd., Bangkok, Thailand, and were average aged 8 weeks with an average body weight of 250 g. The animals were housed in a controlled environment, maintained at a temperature of 24°C ± 1°C with 50% ± 10% relative humidity and a 12:12 h light-dark cycle with light intensities ranging between 250 and 350 Lux. The rats were fed with a standard pelleted diet *ad libitum* and provided with access to drinking water. A 1-week acclimation period was provided before the start of any experimental procedure. Animal procedures were approved by the Walailak University Institutional Animal Care and Use Committee, Thailand (Permit No. WU-ACUC-65064).

### Induction of acute kidney injury

The experiment was divided into 2 groups. The control group consisted of 6 rats that received a 0.9% saline solution (NSS), while the experimental group consisted of 6 rats that received a 20 mg/kg dose of colistimethate sodium. Both groups were given intraperitoneal injections every 12 h continuously for a total of 3 days, with no change in the dosages or frequency of injections being allowed during this period of time.

### Clinical and pathological evaluations

The rats were observed for 7 days to check for any toxic effects, changes in behavior, physical appearance, injuries, pain, and signs of illness. Additionally, their daily water and food intake as well as body weight were monitored (Organisation for Economic Co-operation and Development, 2002). Blood samples were collected from the tail vein on days 0 and 7 to analyze symmetric dimethylarginine (SDMA), blood urea nitrogen (BUN), creatinine, aspartate aminotransferase (AST), alanine aminotransferase (ALT), and alkaline phosphatase (ALP) levels. On day 7, the rats were euthanized, and their vital organs (heart, kidneys, liver, lung, and spleen) were removed and fixed in 10% buffered formalin for subsequent histopathological analysis. The relative kidney weight of each rat was calculated by dividing the kidney weight (in grams) by the body weight of the rat (in grams) and then multiplying by 100.

### Statistical analysis

Descriptive statistics were used to describe data, including percentage, proportion, ratio, and the half-maximal inhibitory concentration (IC_50_), while continuous data were expressed as means and standard deviations (SDs). IC_50_ values of colistimethate sodium on human embryonic kidney cells were calculated by linear approximation regression of the percentage cell viability versus the drug concentration. The unpaired *t*-test was utilized to compare the mean ζ-potential between plain liposomes and CL. Additionally, for each time point, unpaired *t*-tests were used to compare cell viability and IC_50_ values between CS and CL. One-way analysis of variance (ANOVA) for independent samples was performed to compare mean clinical parameters according to three different experimental groups. Two-tailed tests were performed, and a *p*-value of <0.05 was considered statistically significant. Statistical analysis was performed with R statistical software (RStudio, Boston, MA, United States). The graph generation was performed using the commercial software GraphPad Prism (San Diego, CA, United States).

## Results

### Dose-response nephrocytoxicity of colistimethate sodium

**FIGURE 1:**
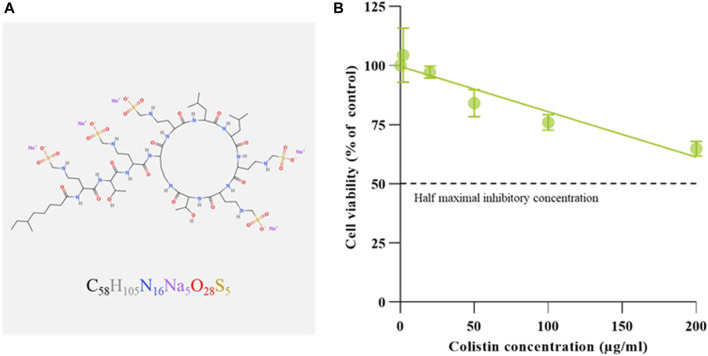
Molecular configuration and renal cytotoxicity of Colistimethate sodium. **(A)** Schematic diagram of chemical structure generated by MolView [(www.molview.org) (Smith, 1995)], along with the simplified molecular-input line-entry system strings obtained from the NCBI PubChem database (National Center for Biotechnology Information, 2023). **(B)** Dose-response curve of colistimethate sodium on human embryonic kidney cells. Percentages of cell viability calculated from cells exposed to with normal saline (control), and 2, 20, 50, 100, and 200 μg/mL of colistimethate sodium. Data represents the mean ± SD of four independent experiments. Half maximal inhibitory concentration (IC_50_) values of CS on human embryonic kidney cells calculated by linear approximation regression of the percentage cell viability versus the drug concentration.

To examine the colistimethate sodium induced cytotoxicity, cell viability for human embryonic kidney cells was determined using MTT reduction assay. The cell viability was represented by the detection of enzyme mitochondrial dehydrogenase activity. The cytotoxic effect of the colistimethate sodium on kidney cell line for 24 h was shown in Figure 1. The results show that colistimethate sodium induced cytotoxicity in a concentration-dependent manner. The viability rate of the kidney cell line from the control group was 100%, while that of the kidney cell line treated with 2, 20, 50, 100, and 200 μg/mL of the colistimethate sodium was 104.34% ± 11.46%, 97.11% ± 2.58%, 84.10% ± 5.70%, 75.96% ± 3.32%, and 64.81% ± 3.13%, respectively. Since, the viability rates of the cells exposed to 200 μg/mL of colistimethate sodium were more than 50% throughout the trial period. However, the IC_50_ or 50% cytotoxic concentration (CC_50_) value was able to calculate by estimated linear regression equation [Y = (−0.1916 × X) + 99.60, *R* ^2^ = 0.8307].

### Liposomal morphology

**FIGURE 2:**
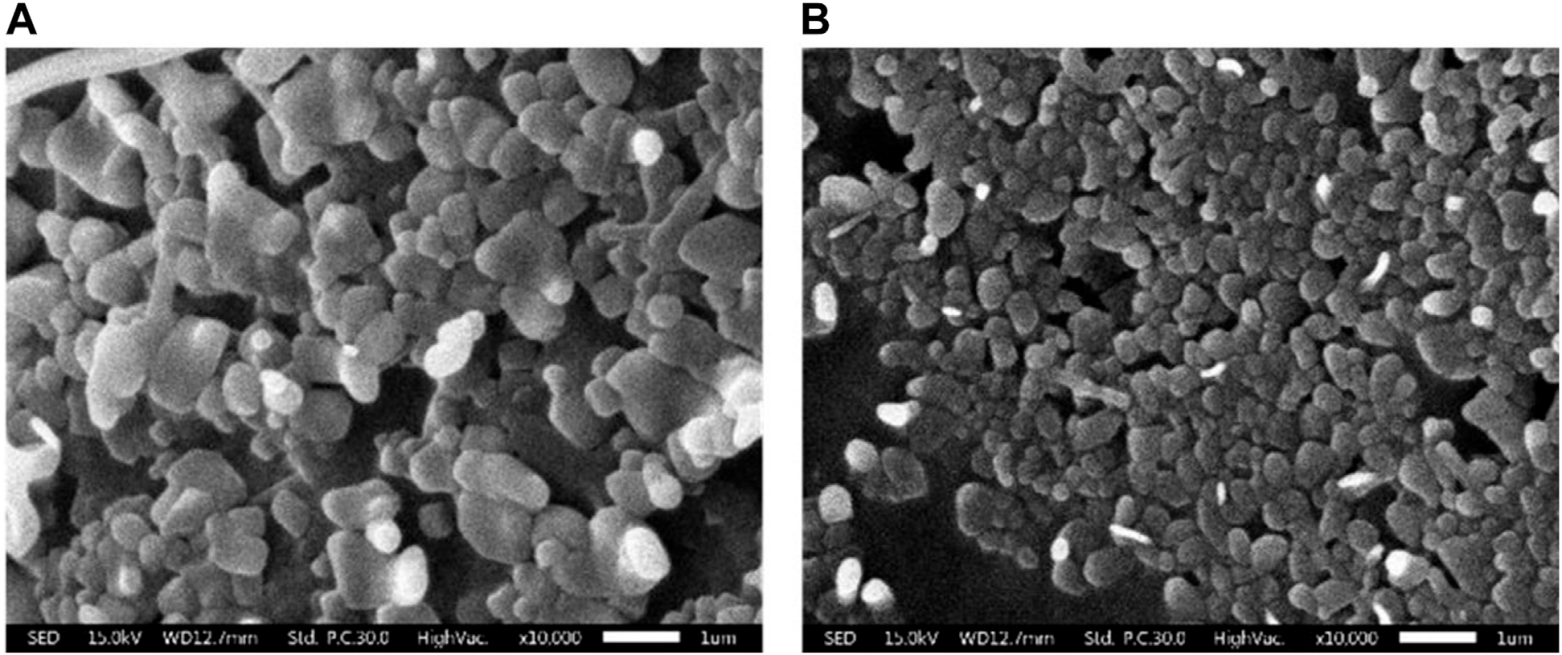
Scanning electron micrographs of liposomal morphology. **(A)** Plain liposome and **(B)** colistin-encapsulated liposome prepared through reverse phase evaporation with a 3:1 ratio of phospholipids to cholesterol.

SEM was utilized to investigate the external morphology of liposomal formation. The SEM image revealed liposomes as spherical structures, which appeared as monodisperse particles with smooth surfaces and distinct boundaries. In particular, the study focused on CL, comparing to plain liposomes. Notably, the micelles within the CL were observed to be smaller than those found in the plain liposomes (Figure 2).

### Liposomal size and stability

**FIGURE 3:**
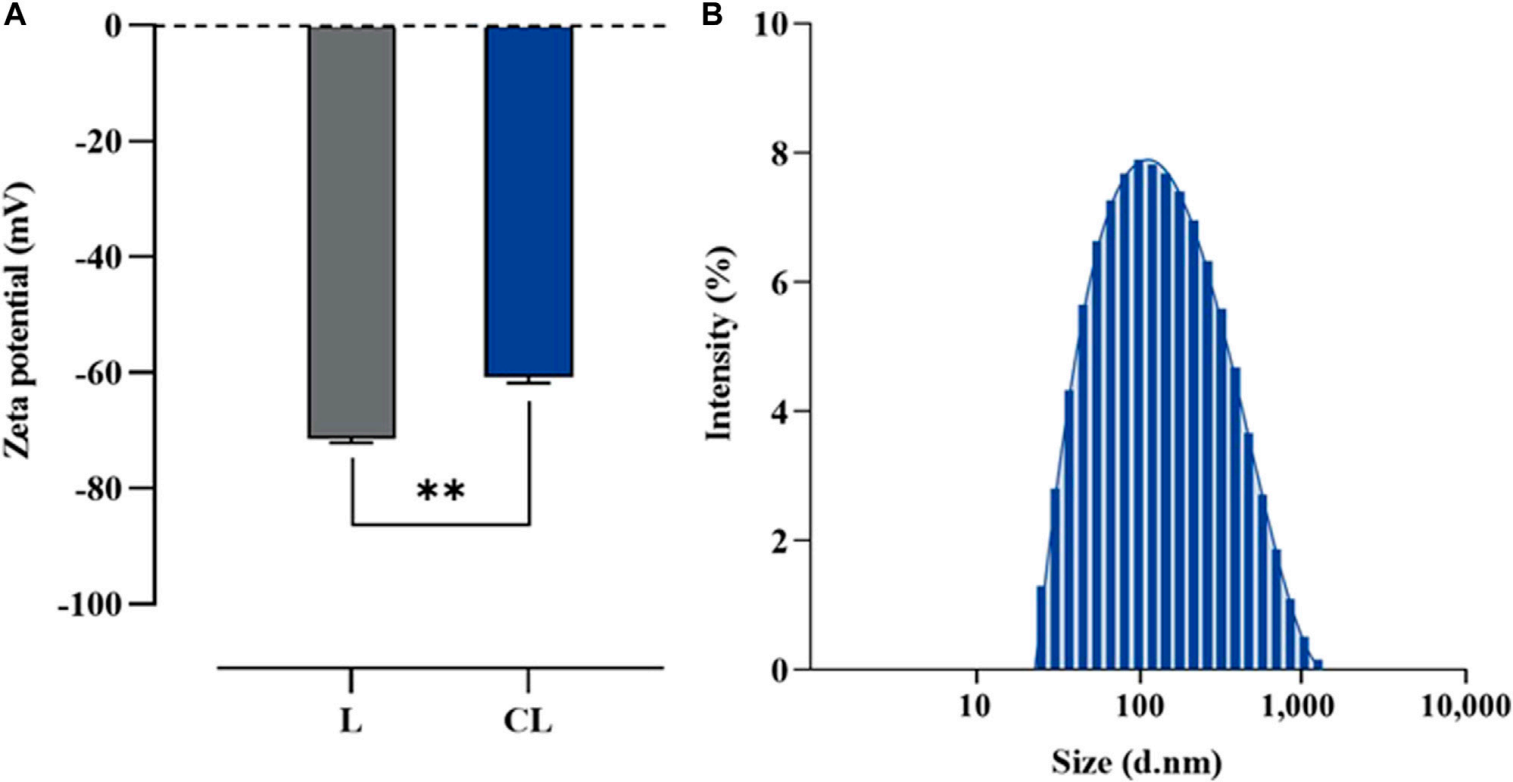
Particular characterization of liposome formulated using a reverse phase evaporation method. **(A)** Z-potential of plain liposome (L) and colistin-encapsulated liposome (CL), and **(B)** Size distribution of CL measured using photon correlation spectroscopy. Horizontal lines with asterisk denote (**) illustrate the significant differences (*p <* 0.01) compared between L and CL preparations, determined through unpaired *t*-tests.

To evaluate the physicochemical properties of the obtained liposomes in terms of size, size distribution and ζ-potential, the dynamic light scattering (DLS) of preparation was also performed using Zetasizer Nano ZS instrument. The result demonstrated that the average size was 151.50 ± 0.46 nm with narrow size distribution of 0.25 ± 0.01 and ζ-potential of −60.80 ± 1.01 mV. Whereas the liposomal preparation without colistimethate sodium had an average size 124.87 ± 2.26 nm with narrow size distribution of 0.44 ± 0.06 and ζ-potential of −71.43 ± 0.64 mV (Figure 3). This finding has important implications for the design and formulation of liposomal drug delivery systems, where balancing the composition of the lipid bilayer is critical for efficient drug delivery.

### Controlled release kinetics of colistimethate sodium

**FIGURE 4:**
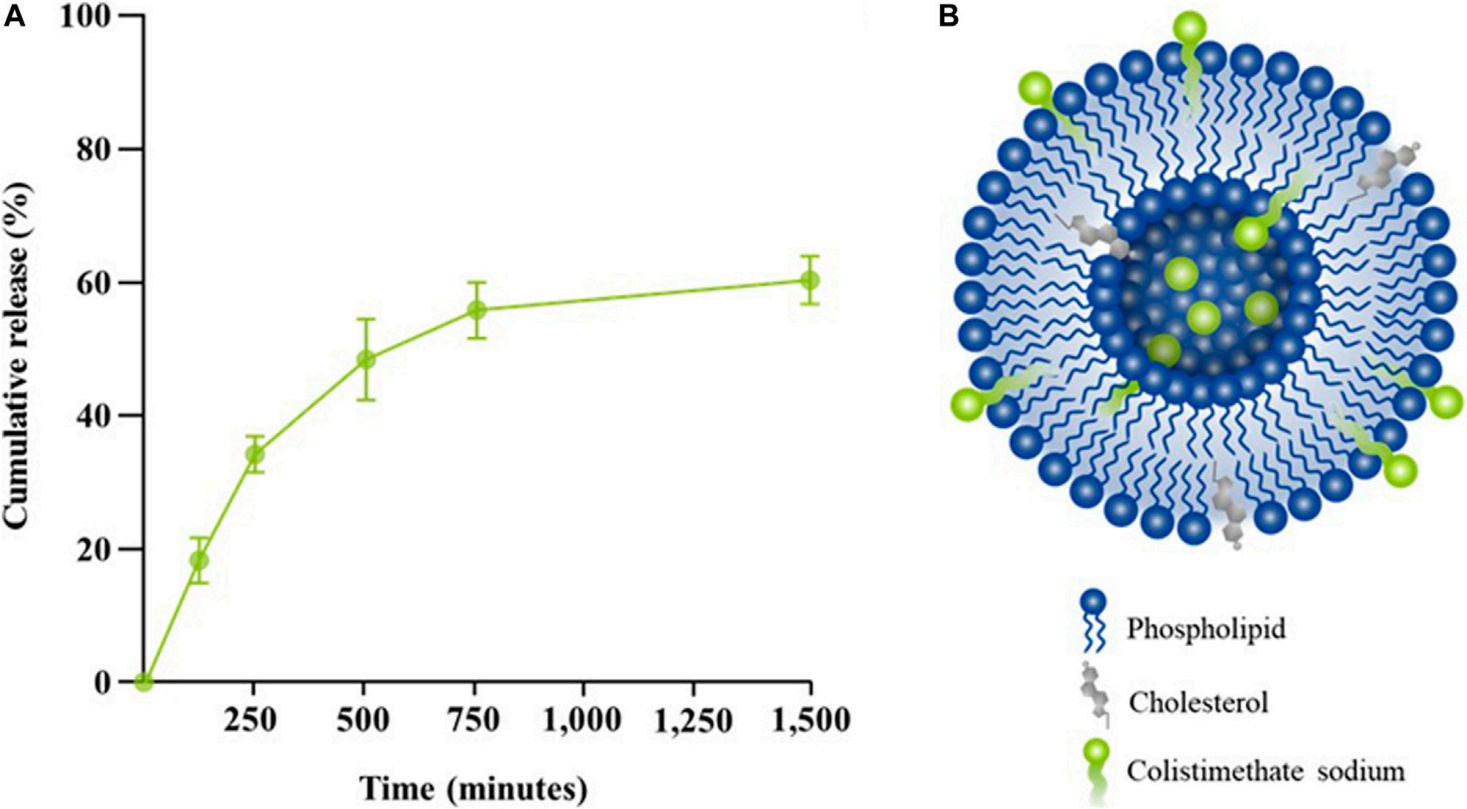
Release kinetic profiles of colistin-encapsulated liposome. **(A)** Percentage of cumulative release of colistimethate sodium from the liposomal formulation measured at 0, 5, 120, 240, 480, 720, and 1,440 min. **(B)** A schematic diagram of colistimethate sodium entrapped within the complex micellar structure of a cholesterol-enhanced liposome carrier system.

Among different molar ratios of a mixture of precise phospholipid and cholesterol, the combination containing 75% phospholipid and 25% cholesterol exhibited the highest drug entrapment efficiency (EE) with an average value of 96.45 ± 0.41. Drug release from liposomes containing colistimethate sodium was measured at 37°C to evaluate the presence of the drug in the PBS release media. It can be seen that the release of colistimethate sodium from the cholesterol-enhanced liposome carrier system occurred in a time-dependent manner. The first 50% release was observed within 480 min, after which it plateaued (Figure 4).

### Time-dependent nephrocytoxicity of the formulation

**FIGURE 5:**
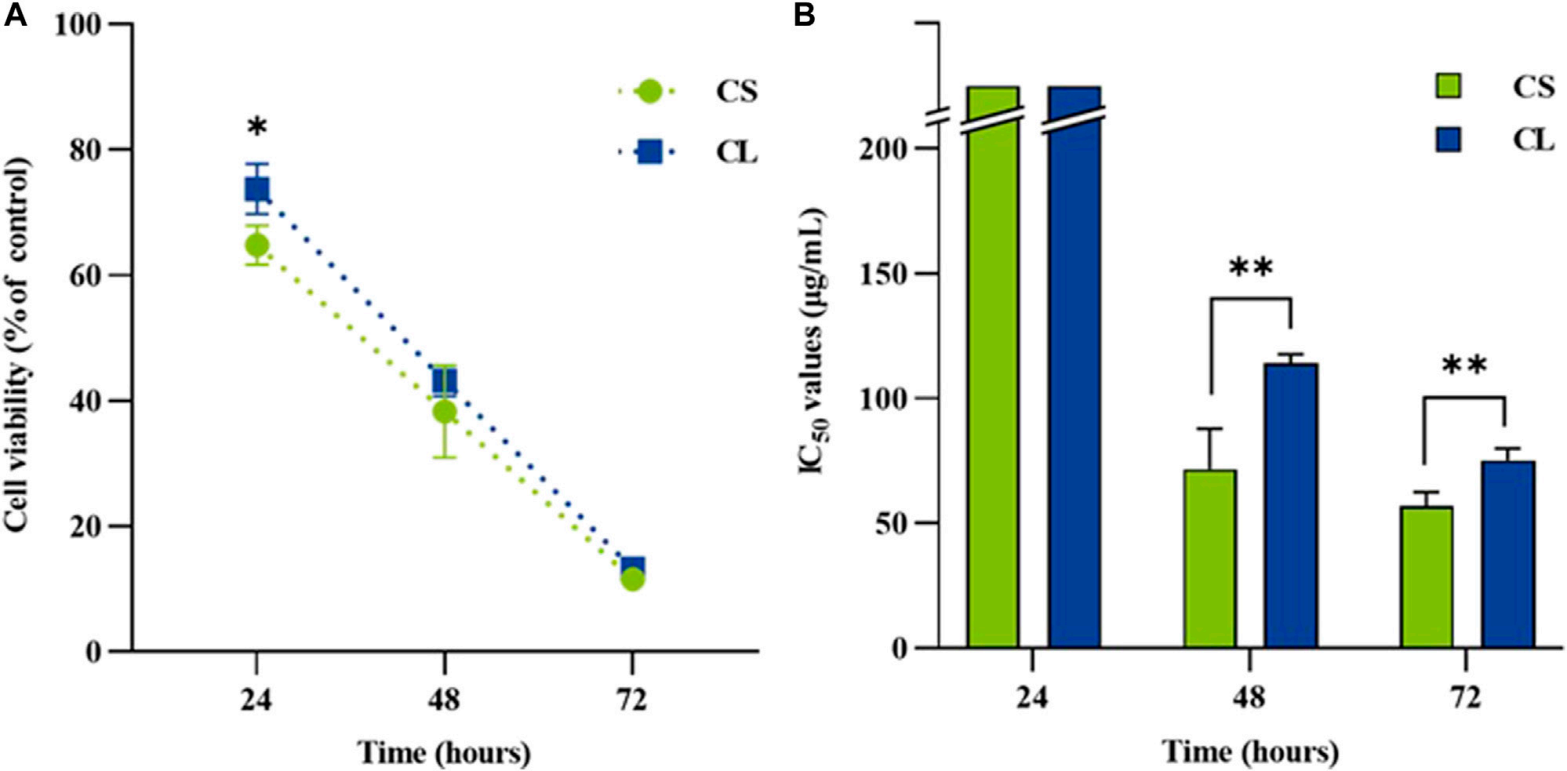
The renal cytotoxicity on human embryonic kidney cells exposed to colistimethate sodium solution (CS), colistin-encapsulated liposome (CL) for 24, 48, and 72 h. For each time point, **(A)** the line chart depicting time-kill kinetics of CS and CL at 200 μg/mL and **(B)** the bar chart displaying the half maximal inhibitory concentration (IC50). The data represents the mean ± SD of four independent experiments, and the analyses employed an unpaired *t*-test. The asterisk (*) indicates a statistically significant difference between CS and CL at *p* < 0.05, while (**) represents significance at *p* < 0.01, both determined through unpaired *t*-tests at each time point.

The study of dose-response cytotoxicity of colistimethate sodium suggested that a value corresponding to the maximum safety concentration on human embryonic kidney cells are given at 200 μg/mL of the colistimethate sodium. Therefore, the cytotoxic effect of the liposome formulations containing 200 μg/mL of the colistimethate sodium on kidney cell line for 24, 48, and 72 h was performed. Figure 5A demonstrated time-dependent killing by colistimethate sodium, while at 24 h, the CL formulation (73.77 ± 3.98) exhibited higher cell viability than the CS formulation (64.81 ± 3.13) (*p* = 0.0375). IC50 values for CS and CL at 24 h could not be estimated due to the concentration limit of 200 μg/mL. However, at 48 and 72 h, CS had significantly lower IC_50_ values (71.67 ± 16.07 μg/mL and 53.33 ± 15.28 μg/mL) than its liposome preparations (114.0 ± 3.61 μg/mL at 48 h, *p* = 0.0112; 118.33 ± 2.89 μg/mL at 72 h, *p* = 0.0019), as shown in Figure 5B.

### Nephrotoxicity of the formulation in rat model

In order to assess the safety of CL, rats received a 20 mg/kg dose of the CL formulation every 12 h for 3 days. The positive control and negative control groups were given CS at the equivalent dose to CL and NSS, respectively. Rat lethality and clinical signs were closely monitored for a period of 7 days. The findings indicated no fatalities in either the NSS or CL groups. However, the CS group exhibited an incident density of fatality at 2.63. The CS group displayed clinical signs of lethargy, disheveled fur, cyanosis, redness, and edema, while no clinical signs were observed in the NSS groups. Intriguingly, in the CL group compared to the CS group, the reduction rates of disheveled fur and edema incident density were 32.47% (*p* = 1.00) and 66.67% (*p* = 0.36), respectively (Table 1).

**TABLE 1:** Incidence density comparison of clinical signs in rats treated with 0.9% saline solution (negative control), 20 mg/kg colistimethate sodium solution, and 20 mg/kg colistin-encapsulated liposome, administered every 12 hours for 3 days.

Clinical signs	Incidence rate (Case number/animal day at risk)			CL baring with NSS			CL baring with CS		
	NSS	CS	CL	δ_abs_	IRR	*p*-value	δ_abs_	IRR	*p*-value
Lethargy	0.00 (0/42)	7.89 (3/38)	0.00 (0/42)	0.00	-	1.00	7.89	-	0.10
Disheveled fur	0.00 (0/42)	7.69 (3/39)	5.13 (2/39)	7.69	0.00	0.23	2.56	1.50	1.00
Cyanosis	0.00 (0/42)	5.00 (2/40)	0.00 (0/42)	0.00	-	1.00	5.00	-	0.23
Redness	0.00 (0/42)	5.13 (2/39)	0.00 (0/42)	0.00	-	1.00	5.13	-	0.23
Edema	0.00 (0/42)	7.69 (3/39)	2.50 (1/40)	2.50	0.00	0.49	5.19	3.08	0.36
Fatality	0.00 (0/42)	2.63 (1/38)	0.00 (0/42)	0.00	-	1.00	2.63	-	0.48

CL, colistin-encapsulated liposome; CS, colistimethate sodium solution; NSS, 0.9% saline solution; IRR, incidence rate ratios; δ_abs_, absolute difference.

Hematological and blood chemistry parameters were compared among the experimental rat groups at day 7 post-administration. Acute kidney injury was successfully induced in the CS group, as evidenced by a significantly elevated serum level of symmetric dimethylarginine (SDMA) (15.00 ± 1.63 μg/dL) compared to the NSS group (10.67 ± 0.47 μg/dL) (*p* < 0.01). Conversely, no significant difference in serum SDMA levels was observed between the NSS and CL groups (8.33 ± 1.70 μg/dL). Moreover, the mean value of total white blood cell count (WBC) was also not different in the NSS group (7.37 ± 0.47 log_10_ cells/mL) and the CL group (7.29 ± 0.99 log_10_ cells/mL). In the CS group, WBC levels was 9.73 ± 0.51 log_10_ cells/mL, which were significantly higher than those of the NSS and CL groups (*p* < 0.01). However, no significant differences were observed in other blood parameters (Table 2).

**TABLE 2:** Comparison of rat blood parameters at 7 days post-administration of 0.9% saline solution (negative control), 20 mg/kg colistimethate sodium solution, and 20 mg/kg colistin-encapsulated liposome, administered every 12 h for 3 days.

Blood parameter	Experimental groups			p-value
	NSS	CS	CL	
RBC (log10 cells/mL)	7.21 ± 0.24	7.48 ± 0.32	7.52 ± 0.57	0.62
Hb (g/dL)	14.05 ± 0.50	14.40 ± 0.27	14.68 ± 1.04	0.56
Hct (%)	42.33 ± 1.54	44.00 ± 0.19	45.03 ± 2.48	0.19
WBC (log10 cells/mL)	7.37 ± 0.47^a^	9.73 ± 0.51^b^	7.29 ± 0.99^a^	<0.01
PLT (log10 cells/mL)	805.00 ± 121.15	801.50 ± 68.06	780.00 ± 73.09	0.97
Cr (mg/dL)	<0.10 ± 0.00	<0.10 ± 0.00	<0.10 ± 0.00	N/A
BUN (mg/dL)	27.75 ± 2.28	23.25 ± 3.49	25.00 ± 3.00	0.23
ALT (U/L)	88.00 ± 17.85	97.75 ± 9.78	75.75 ± 30.89	0.48
AST (U/L)	207.00 ± 34.26	218.00 ± 27.90	166.50 ± 57.78	0.33
ALP (U/L)	272.75 ± 20.14	255.25 ± 30.96	231.00 ± 12.69	0.13
GGT (U/L)	4.00 ± 1.22	1.25 ± 1.64	6.50 ± 4.56	0.18
SDMA (µg/dL)	10.67 ± 0.47^a^	15.00 ± 1.63^b^	8.33 ± 1.70^a^	<0.01

^a,b^Distinctive superscript letters shared within the same row are significantly different (*p* < 0.05). ALP, Alkaline phosphatase; ALT, Alanine transaminase; AST, Aspartate transaminase; BUN, Blood urea nitrogen concentration; CL, Colistin-encapsulated liposome; Cr, Creatinine; CS, Colistimethate sodium solution; GGT, Gamma-glutamyl transferase; Hb, Hemoglobin concentration; Hct, Hematocrit; NSS, 0.9% saline solution; PLT, Platelet count; RBC, Red blood Cell Count; SDMA, Symmetric dimethyl arginine; WBC, White blood cell count.

**FIGURE 6:**
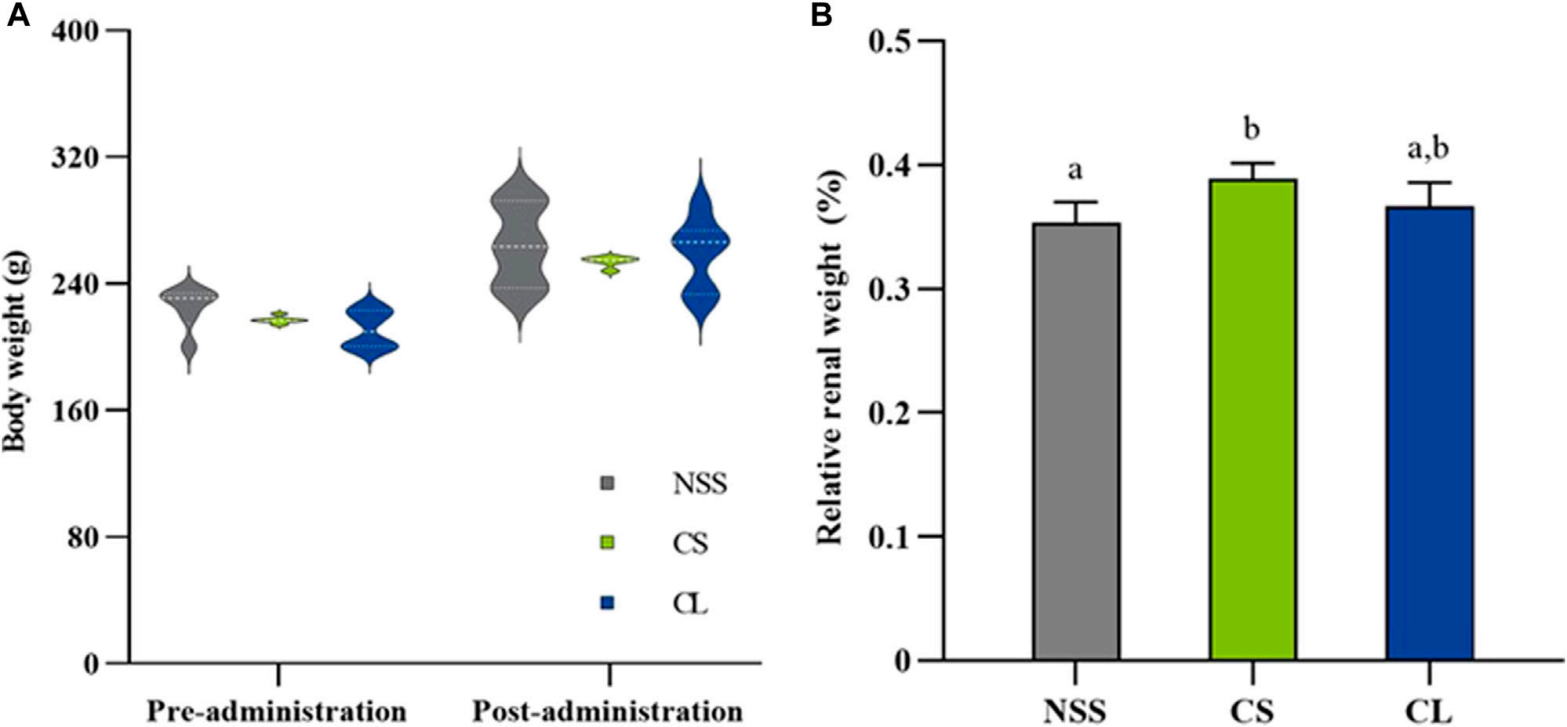
Comparison of body and renal weights in rats treated with 0.9% saline solution (NSS), 20 mg/kg colistimethate sodium solution (CS), and 20 mg/kg colistin-encapsulated liposome (CL), administered every 12 h for 3 days. **(A)** Mean body weights at pre-administration (day 0) and post-administration (day 7) and **(B)** relative renal weights post-administration of the three experimental groups. The data underwent analysis through a two-way ANOVA followed by Tukey’s multiple comparison test. Bars without a common letter indicated significant differences (*p <* 0.05).

During 7 observation days, the mean body weight did not differ significantly among the three experimental groups (Figure 6A). Additionally, the calculation of average daily gain (ADG) for the NSS (7.94 ± 1.47 g), CS (7.53 ± 0.65 g), and CL (7.69 ± 1.20 g) groups showed no significant differences. The results revealed that the CS group (0.39 ± 0.01 g) exhibited a greater relative renal weight after 7 days compared to the NSS group (0.35 ± 0.02 g) (*p <* 0.05). However, CS group relative renal weight was, which were not significantly different from NSS and CL (0.37 ± 0.02 g) groups (Figure 6B).

**FIGURE 7:**
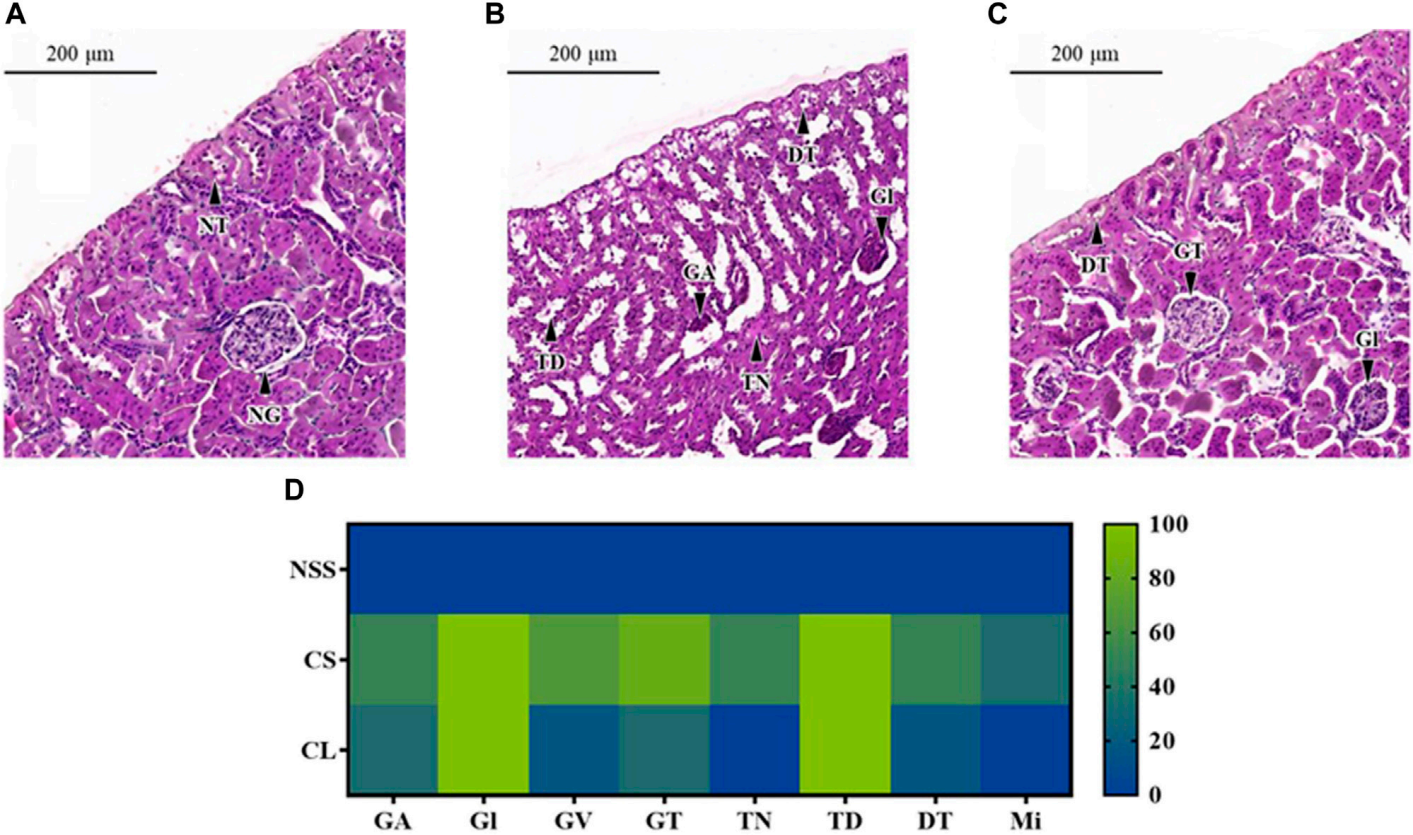
Microscopic characterization of rat renal tissues. Photomicrography of representative renal cross section with hematoxylin and eosin staining from rats after administrated with **(A)** 0.9% saline solution (NSS), **(B)** 20 mg/kg colistimethate sodium solution (CS), and **(C)** 20 mg/kg colistin-encapsulated liposome (CL) every 12 h for 3 days. **(D)** The heat map illustrated the occurrence percentage of histopathological renal lesions found in each individual kidney relative to the total number of kidneys. Features noted here include GA, Glomerular atrophy; DT, Degenerative renal tubules; Gl, Glomerulitis; GT, Glomerular basement thickening; GV, Glomerular vacuolation; Mi, Mineralization; NG, Normal glomerulus; NT, Normal renal tubules; TD, Tubular dilatation; TN, Tubular necrosis.

The microscopic examination of rats in the NSS group reveals normal glomerular and renal tubular structures (Figure 7A). Conversely, histopathological renal lesions are observed in the CS and CL groups, as shown in Figures 7B, C, respectively. In addition, the occurrence percentage of these histopathological renal lesions in each individual kidney, relative to the total number of kidneys, was illustrated in Figure 7D.

In both the CS and CL groups, full occurrence rate of Glomerulitis (Gl) and tubular dilatation (TD) were observed. The percentage of glomerular basement thickening (83.33%), glomerular vacuolation (66.67%), glomerular atrophy (50.00%), and degenerative renal tubules (50.00%) was higher in the CS group compared to the CL group, which had incidence rates of 33.33%, 16.67%, 33.33%, and 16.67%, respectively. The CS group had a half occurrence rate of tubular necrosis (TN) and a one-third occurrence rate of mineralization (Mi), whereas these pathological changes were absent in the CL group.

## Discussion

The colistin is composed of both hydrophobic and hydrophilic regions. The fatty acyl chain at the N-terminal end constitutes the hydrophobic region, responsible for interacting with the bacterial membrane. Meanwhile, the cyclic heptapeptide ring constitutes the hydrophilic region, imparting colistin with its antimicrobial activity (Ayoub Moubareck, 2020). In this study, CL were prepared using the reverse phase evaporation method with a 3:1 ratio of phospholipids to cholesterol. Our results also demonstrate that the CL had approximately a size of 150 nm, a ζ-potential of −60 mV, and an EE of 95%. While the size was consistent with the previous experiment, the EE showed improvement compared to the earlier findings (Wallace et al., 2012; Li et al., 2016; Menina et al., 2019). The CL morphology from SEM images is also consistent with previous research conducted on liposomes prepared through the lipid film hydration method (Menina et al., 2019). Our preparation method is particularly suitable for colistin as it has the advantage of being able to encapsulate both hydrophilic and hydrophobic drugs. This is because liposomes are formed by a water-in-oil emulsion, which allows for the incorporation of both water-soluble and lipid-soluble compounds (Akbarzadeh et al., 2013; Lu and Qu, 2021). This finding aligns with previous research which average size of colistin-loaded liposomes, prepared using dioleoylphosphatidylcholine with or without cholesterol at a molar ratio of 2:1, to be around 180 nm (Wallace et al., 2012). Furthermore, previous research has also suggested that using a 70:30% ratio of lipids to cholesterols is optimal for increasing stability without altering the lipid composition, while ensuring controlled and reproducible drug release (Briuglia et al., 2015). The presence of cholesterol to liposomes can increase in liposome size (Choi et al., 2023) and have significant effects on liposomes stability fluidity, permeability, and stability. This effect is due to ability of cholesterol to increase the packing density of phospholipids in the bilayer and may interact with phospholipids through hydrogen bonding (Pandit et al., 2004), leading to increased membrane rigidity and decreased fluidity that can affect permeability (Lombardo and Kiselev, 2022).

The cell-based toxicity assay on human embryonic kidney cells was performed for prognosticating toxicity of colistin prior to study in the killing kinetic of the formulations. The cytotoxicity result of the CS on human embryonic kidney cells at 24 h after exposure was in dose-dependent manner. The percentage of cytotoxicity at highest concentration of the colistin (200 μg/mL) is lower the half maximal inhibitory concentration this study. These findings are in agreement with previous study on colistin-induced renal proximal tubular cells (RPTEC/TERT1) toxicity, which the IC_50_ value of colistin was at concentrations above 200 μg/mL (Worakajit et al., 2021). The median toxic concentration (TC_50_) of 70 μM colistin on human proximal tubule kidney cell line (HK-2) upon 24-h treatment was also previously documented (Keirstead et al., 2014). Whereas the previous study demonstrated that cytotoxicity of colistin has weak toxic activity on human erythrocyte (Mohamed et al., 2016). The effects of colistin in combination on Vero cells were also toxic at 1 mg/mL (Naghmouchi et al., 2013). These results suggest that 200 μg/mL colistin has strong safety on the kidney cells exposed directly. The MTT assay is the commonly applied for evaluating of cytotoxicity for screening the drugs (Berridge et al., 2005). In this assay, the reduction of MTT is linked to the metabolic activity of intracellular reductases, including mitochondrial dehydrogenase (Rai et al., 2018). While this method has limitations in elucidating the mechanisms of colistin-induced kidney cell death, previous research suggests that colistin causes mitochondrial and endoplasmic reticulum dysfunction (Gai et al., 2019).

Exposure of human embryonic kidney cells to the colistin formulations reduced cellular metabolic activity concentration and time-dependently. Moreover, the liposome formulation of colistin (IC_50_ > 200 μg/mL) had unacceptable cytotoxicity at 24 h. Interestingly, the viability of human embryonic kidney cells after exposure with colistin solution was higher than that of their liposome preparation. These results indicated that the liposome preparations of colistin had biocompatibility and low toxicity. Overall, our results are in accordance with previously reported cytotoxicity of the liposomal amphotericin B studied (Roberts et al., 2015). It has been hypothesized that the nephrocytotoxicity of colistin in liposomal form is lower than that of unencapsulated colistin. Since, liposomes are tiny vesicles that encapsulate nephrotoxic drugs and are made of phospholipid bilayer. It may have a higher therapeutic index and less toxicity since the reticuloendothelial system and macrophages can absorb it preferentially (Ameen, 2017). In addition, the liposomal drug-delivery systems offer a very interesting opportunity for delivering drugs with reduced nephrotoxicity. However, *in vivo* nephrotoxic effect of the colistin formulations needs to be investigated for the best possible therapeutic approach.

In a nephrotoxicity study, rats were given 20 mg/kg of colistin every 12 h for 3 days and compared to a negative control group. The results showed clinical signs of toxicity, including disheveled fur, lethargy, redness, edema, cyanosis, and fatality. Although the levels of BUN and Cr in the CS group remained unchanged, the study also found significantly elevated levels of WBC, and all histopathology lesions in the central nervous system were associated with acute kidney injury (Heybeli et al., 2019). The results of this study were consistent with previous studies that used 20-mg/kg/8-h, 30-mg/kg/12-h, and 150-mg/kg/12-h colistin methanesulfonate via a jugular vein cannula for 7 days (Wallace et al., 2008), as well as another study that used 12–36 mg/kg/day colistimethate sodium intramuscular injection every 12 h for 7 days (Ghlissi et al., 2013). Since, the development of colistin-induced nephrotoxicity can be attributed to the binding of colistin to the cell membrane of glomerular and proximal tubule cells, which results in an increased membrane permeability and the loss of water and ions from the cells. This process can lead to kidney damage, as evidenced by previous studies (Ordooei Javan et al., 2015; Petejova et al., 2019). The results of this experiment further support the potential risk of acute kidney injury, as shown by the significant increase in SDMA levels. This finding is consistent with previous research that demonstrated the effectiveness of serum SDMA as a biomarker of renal excretory function in a rat model of gentamicin-induced proximal tubular injury, as well as the validation of a high-throughput SDMA immunoassay for rat serum (Hamlin et al., 2022).

Interestingly, the reduction in all clinical signs, WBC count, SDMA levels, and histopathological lesions was observed in the CL group. These results suggest that the liposome formulation can effectively protect against the nephrotoxic effects of colistimethate sodium, which is consistent with previous studies on doxorubicin (Gabizon et al., 2003), cisplatin (Uchino et al., 2005), polymyxin E sulfate (Wang et al., 2009), amphotericin B (Stone et al., 2016), and vancomycin (Joshi et al., 2023). The precise mechanism by which liposomes reduce colistin nephrotoxicity is not yet fully understood, although several possibilities have been proposed. One possibility is that liposomes interact with cells through various mechanisms. When liposomes come into contact with cells, they can merge with the cell membrane and release their contents into the cell. This characteristic can be beneficial for delivering drugs or other therapeutic agents directly to the interior of cells (Pei and Buyanova, 2019). Additionally, liposomal encapsulation enhances the stability and safety of antimicrobials, resulting in more appropriate pharmacokinetic and pharmacodynamic profiles by prolonging the circulation time in the bloodstream (Ferreira et al., 2021). The results suggest that liposomal drug delivery systems can effectively reduce both *in vitro* and *in vivo* nephrotoxicity. In addition, this information can be used to improve patient outcomes through the use of liposomal colistin medication. Further clinical studies are warranted to determine the best possible safety approach.

## Conclusion

This study has highlighted that the preparation of colistin-encapsulated liposomes is successful and plays an important role in ensuring effective pharmaceutical properties. Additionally, the use of liposomes has been found to protect human embryonic kidney cells from concentration- and time-dependent cytotoxicity. Notably, the liposomal formulation of colistin has also been found to particularly decrease clinical and pathological nephrotoxicity in rat models, which underscores their potential in enhancing safety. Overall, these findings provide a comprehensive understanding of the benefits of liposomal systems and emphasize the need for further research into optimizing drug delivery systems for use in human clinical studies.

## Data Availability

The original contributions presented in the study are included in the article/Supplementary material, further inquiries can be directed to the corresponding authors.
